# Emergency access to the subclavian vessels by non-thoracic surgeons: a cadaver-based learning model for orthopedic trauma surgery

**DOI:** 10.1007/s00402-026-06367-4

**Published:** 2026-06-03

**Authors:** Peter Grechenig, Axel Gänsslen, Jan Dauwe, Ulrike Wittig, Maximilian Sagmeister, Amir Koutp, Paul Puchwein, Patrick Sadoghi, Gloria Hohenberger

**Affiliations:** 1 Medical University of Graz, Graz, Austria; 2 Klinikum Wolfsburg, Wolfsburg, Germany; 3 University Hospitals Leuven, Leuven, Belgium; 4 Wiener Neustadt State Hospital, Wiener Neustadt, Austria; 5 State Hospital Feldbach-Fürstenfeld, Feldbach, Austria; 6 Danube Private University, Krems, Austria

**Keywords:** Emergency access, Learning curve, Subclavian vessels, Subclavian artery, Subclavian vein, Anatomical dissection course, Thoracic injuries

## Abstract

**Purpose:**

The aim of this study was to evaluate the procedural time and safety of an infraclavicular approach to the subclavian vessels for damage control of major upper extremity vascular injuries. The study specifically focused on the performance of non-thoracic surgeons using a cadaveric model.

**Methods:**

A sample of 100 orthopedic trauma surgeons (residents, specialists, and attendings) was recruited from an AO cadaveric dissection course. Emergency infraclavicular access was performed on 50 cadavers over two consecutive days. The procedural time, successful vessel clamping, participant self-assessment, and the resulting learning curve were analyzed.

**Results:**

On day one, 27% (9/33) of participants who successfully achieved correct clamping of both subclavian vessels reported feeling confident. By day two, this proportion increased to 71% (55/77). Comparing day 1 to day 2 we found an improvement of 35% (subclavian artery) and 37% (subclavian vein) in the correct identification of subclavian vessels in our sample. The evaluations of this study show that there is no correlation between surgical experience and successful emergency access.

**Conclusion:**

Anatomic dissection is of paramount importance for teaching rare and demanding surgical techniques. This study demonstrates that anatomical workshops significantly improve procedural safety and self-assessment when accessing subclavian vessels during emergencies.

## Background

Subclavian artery injuries represent rare traumas accounting for 1 to 2% of all vascular injuries [1, 2]. Their reported incidence is 0.4% in thoracic injuries and rises to 3.0% in penetrating neck and chest injuries [3, 4].

In approximately 20% of all cases, both the subclavian artery and vein are injured. Concomitant lesions of the brachial plexus occur in one third of all patients [4].

Subclavian artery injuries are associated with a high mortality rate and 20% of all patients reach the hospital without vital signs due to hemorrhagic shock [2–5].

Therefore, these injuries demand rapid diagnostics and treatment strategies. Subclavian vessel traumas are challenging since their surgical exposure has been reported as difficult even for experienced surgeons [6, 7]. However, for orthopedic and trauma surgeons, attaining proximal vascular control is paramount in major complex injuries to the upper extremities. Fracture dislocation near the shoulder joint with neurovascular injuries, e.g. four-quarter amputation or complex fracture dislocation of the shoulder frequently manifest with significant vascular involvement requiring immediate damage control vascular procedures [8].

Hemorrhage continues to represent one of the most preventable causes of early death in trauma patients. While structured trauma care systems and standardized protocols such as ATLS^®^ have significantly improved early management and prioritization of life-threatening injuries, definitive surgical hemorrhage control in anatomically complex regions remains technically demanding [9–11].

The current medical literature remains sparse regarding on how to effectively train junior surgeons in these time-critical and rapid procedures. Cadaveric workshops are one of the options to improve performance of surgical skills and both trainees and teachers see them as highly effective for skill acquisition [12].

The primary aim of this study was to evaluate procedural safety and the learning curve associated with emergency infraclavicular exposure of the subclavian vessels performed by non-thoracic surgeons in a cadaveric training model. Specifically, we assessed accuracy of vessel identification, procedural time, occurrence of potentially harmful errors, length of skin incision, and correct emergency access including clamping. A secondary aim was to determine whether procedural performance correlated with clinical seniority or prior surgical experience and to evaluate changes in self-assessed confidence following repeated anatomical training.

## Methods

### First day

**Fig. 1:**
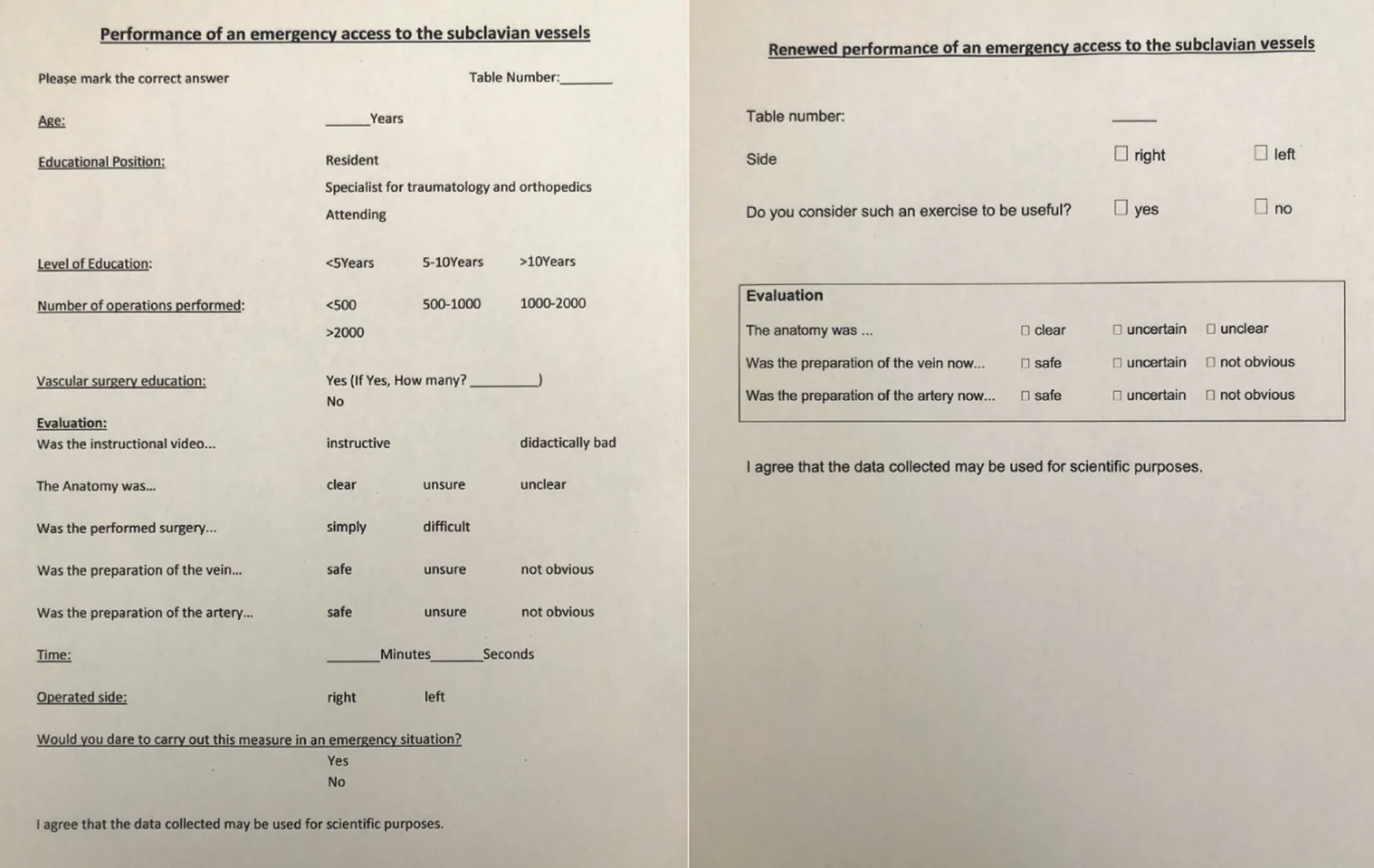
Evaluation form of day 1 and 2

A sample of 100 residents, specialists and attendings for orthopedic trauma was enrolled during a cadaver dissection course of the “Arbeitsgemeinschaft für Osteosynthesenfragen (AO)” on extremity approaches. Based on a questionnaire, the individual surgical experience was analyzed based on experience in damage control vascular surgical experience, individual experience etc. (Fig. 1). Written informed consent was obtained from all participants. Fifty Thiel-embalmed human cadavers were utilized in the current study [13].

**Fig. 2:**
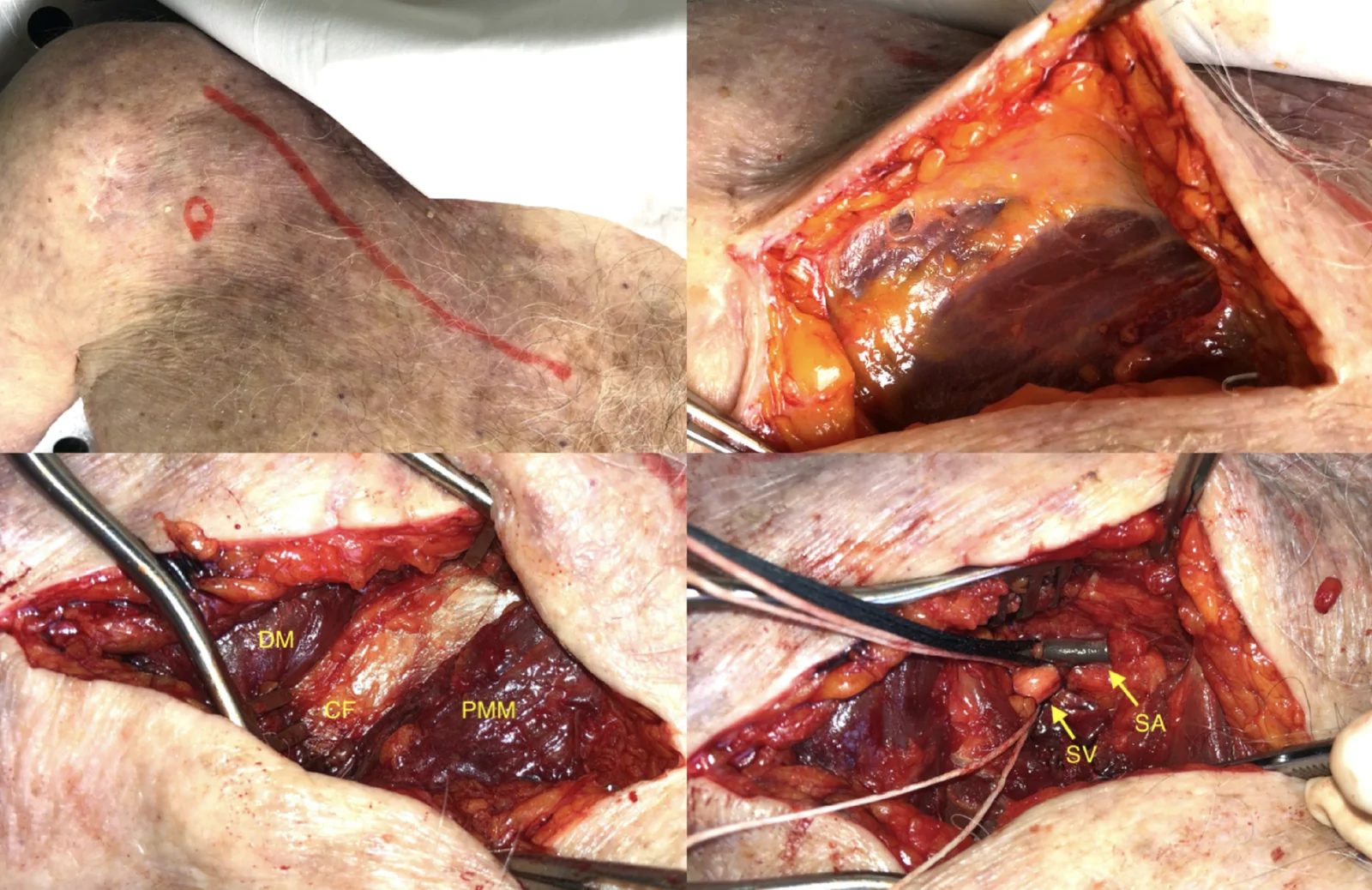
Showing a step-by-step way of performing an infraclavicular approach to the right subclavian vessels. *DM* deltoid muscle, *CF* clavipectoral fascia, *PMM* pectoralis major muscle, *SV* subclavian vein, *SA* subclavian artery. For demonstration, the black loop attached the subclavian artery and the white loop the subclavian vein

A demonstrating video on how to perform an infraclavicular access to the subclavian vessels was shown to all participants prior to the procedure. The surgical procedure was shown in a systematic step-by-step approach (Fig. 2), including:

- Anatomic demonstration of the infraclavicular region by an anatomist,
- Identification of the infraclavicular groove,
- Identification of the subclavian vessels,
- Preparation and fixation of the subclavian vessels with different colored loops.

**Fig. 3:**
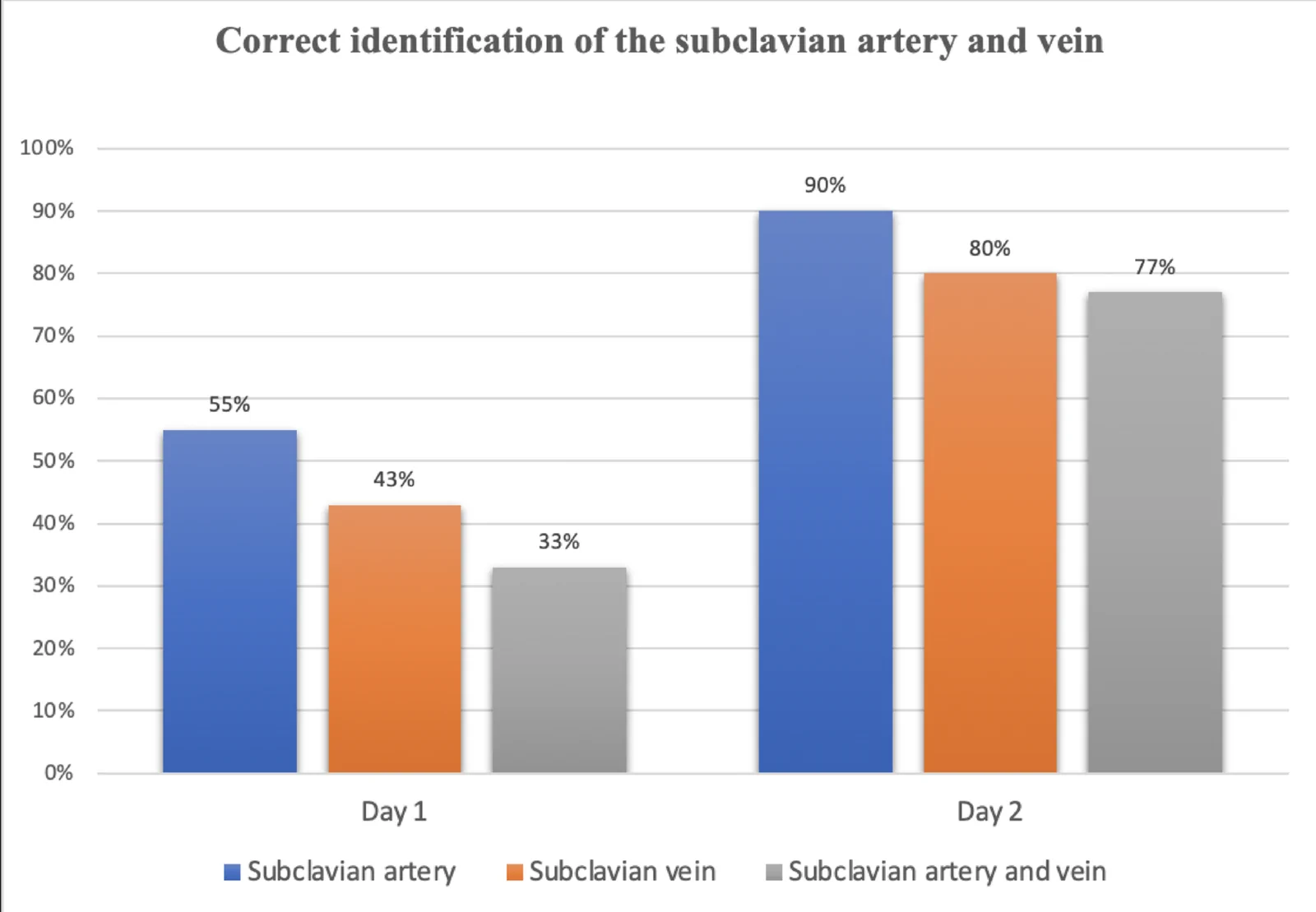
Comparing the progress of performance from day one to day two

The cadaver was in supine position with a roll between the shoulder blades fixed on a dissecting table. Furthermore, each of the surgeons had an assistant as a course participant for the operation (see Fig. 3).

### Anatomy

The subclavian artery is located posterior and slightly cephalad in the surgical field than the subclavian vein. After identification of the subclavian vein, the vein was mobilized and fixed with a loop. Further dissection was performed to identify the subclavian artery with mobilization and looping. The time from start of the skin incision until definitive placement of both loops was measured and the anatomical accuracy of the procedure and potentially harmful complications were analyzed by an experienced trauma surgeon and an anatomist.

During the preparation, it was emphasized several times that this was an emergency procedure and time was also stopped. Potential complications included: injuries to the subclavian and/or cephalic vein, to the subclavian artery and the brachial plexus.

### Second day

On the subsequent day the instructional video was re-screened, and the approach and anatomy were explained and demonstrated in detail on the specimens. The participants then were instructed to correctly identify and loop the artery and subclavian vein on their approach from the previous day. This enabled them to correct their access and identification once again. A questionnaire was again evaluated as the previous day (Fig. 1).

After the exercise, the anatomical accuracy of the procedure and potential harmful complications were analyzed by an experienced trauma surgeon and an anatomist.

The level of education was divided in either resident, specialists (surgeons after finished residency with 3 or less years of experience) and attendings (surgeons being specialists for at least 3 years or more).

### Statistical analysis

In addition to descriptive summary of data, statistical analysis was performed using Statistical Package for the Social Sciences (SPSS) version 27.0 software (IBM Corp., Armonk, NY, USA). In order to quantify potentially significant differences regarding the correct identification of the subclavian artery, the subclavian vein, and potential complications between day 1 and day 2 of dissection, the Chi squared test was performed. Moreover, Chi squared tests were conducted in order to identify significant differences between residents, specialists and attendings regarding correctness of incision, correct identification of artery and vein, complications and courage, respectively. ANOVA analysis was used when testing for statistical significance concerning the length of the incision and if dissection was performed by a resident, specialist or attending. A value of *p* < 0.05 was set to show statistical significance.

### Compliance with ethical standards

All studies on human cadaver are approved by the institutional review board of the Medical University, Department of Anatomy under the law of donation. The study was performed in accordance with the relevant guidelines and regulations.

## Results

A total of 100 emergency approaches were executed on 50 cadaveric specimens. The mean age of the participants was 31.5 years (SD 5). Regarding clinical seniority, 84 participants were residents, 11 were specialists and 5 were attendings.

Seventy participants had less than 5 years of experience in orthopedic trauma surgery, 29 had between 5 and 10 years, and 1 participant had more than 10 years of experience. With respect to the number of performed surgeries, 80 participants had performed fewer than 500 operations, 13 had performed 500–1000 operations, and 7 had performed 1000–2000 operations. None had performed more than 2000 operations. 6 participants reported previous vascular surgery training, while 94 had no such experience. The mean number of previously performed vascular procedures among those trained was 23.

### First day

Ninety-six participants reported that the anatomy video shown at the beginning was instructive, whereas four participants rated it as didactically poor.

The anatomy of the emergency access and surrounding structures was considered clear by 63 participants, 35 participants felt uncertain during preparation, and 3 participants reported that the anatomy was not clear. Forty-eight participants perceived the procedural complexity as low, while 52 experienced it as difficult.

Fifty participants stated they were confident regarding correct looping of the subclavian vein, 46 were unsure, and in 4 cases vein occlusion was not achieved. Regarding arterial looping, 55 participants felt confident, 42 were unsure, and in 3 cases the artery was not clamped.

Thirty-eight participants reported that both artery and vein were easy to identify. Correct access with successful identification and clamping of both vessels was achieved by 33 participants.

The mean duration of the emergency access from skin incision to successful clamping of artery and vein was 7 min and 46 s (SD 3 min and 36 s).

Sixty participants reported confidence in their ability to perform such an operation, whereas 40 did not feel confident.

Evaluation by an anatomist and a trauma surgeon revealed a mean incision length of 7.57 cm (SD 1.91 cm). Ninety-six participants chose the correct access, while 4 missed the appropriate approach. Overall, complete and correct emergency access to the subclavian vessels, including clamping, was achieved by 33 participants; 67 participants did not achieve a fully successful result.

Correct arterial looping was achieved by 55 participants. In unsuccessful cases, the subclavian vein was clamped 17 times, and the brachial plexus was wrapped in 28 cases.

Correct looping of the subclavian vein was achieved by 43 participants. In unsuccessful cases, the subclavian artery was incorrectly clamped 13 times, the brachial plexus 25 times, and the cephalic vein 19 times.

Among the six participants with vascular surgery training, only one achieved correct access with successful clamping of both vessels. Two participants chose the wrong approach, clamping the brachial plexus instead of the artery and the artery instead of the vein. Of the 33 participants who successfully achieved correct clamping of both subclavian vessels on day one, 27% (9/33) reported feeling confident.

No significant differences were found between residents, specialists, and attendings regarding incision length (*p* = 0.513; mean values: 7.7 cm, 7.1 cm, and 7.0 cm, respectively) or correct approach rate (*p* = 0.609). Correct identification of the subclavian artery on day one was achieved by 46 residents, 8 specialists, and 2 attendings, with no significant difference between groups (*p* = 0.383). Correct identification of the subclavian vein showed no significant group differences (*p* = 0.274). No differences were observed regarding complications (*p* = 0.728) or self-reported confidence (*p* = 0.394).

### Second day

On the second day, 91 participants rated the anatomy video as clear, 8 as uncertain, and 1 as unclear. Ninety-nine participants considered the repeated exercise useful.

Eighty-three participants felt comfortable identifying the subclavian artery, 15 were uncertain, and 2 were unsure. For identification of the subclavian vein, 74 participants felt comfortable, 24 uncertain, and 2 unsure.

Re-evaluation by the anatomist and trauma surgeon showed that 77 participants correctly performed emergency access to the subclavian vessels, including clamping. Seventy participants reported that both artery and vein were easy to identify, and 77 participants correctly identified and clamped both vessels. Of the 77 participants who successfully achieved correct clamping of both subclavian vessels on day two, 71% (55/77) reported feeling confident.

Correct identification of the subclavian artery increased from 55 participants on day one to 90 participants on day two (*p* = 0.01). Correct looping of the subclavian vein increased from 43 to 80 participants (*p* = 0.01). Significantly fewer complications were observed on day two compared to day one (*p* = 0.01). None of the participants who correctly looped both vessels on day one made an incorrect decision on day two (see Table 1).

**Table 1:** Outcomes day 1 compared to day 2

Assessment	Day 1	Day 2
Anatomy video rating (n=%)	96% Clear 4% Uncertain	91% Clear 9% Uncertain
Correct identification of Artery	55%	90%
Procedural complexity	48% Easy	70% Easy
Correct performed emergency access to the subclavian vessels, including clamping	33%	77%

## Discussion

The key finding of this cadaver-based study is the pronounced learning curve associated with emergency infraclavicular exposure of the subclavian vessels. Procedural accuracy, correct vessel identification, and participant confidence improved significantly after repeated, structured anatomical training. Importantly, no correlation was observed between clinical seniority or prior surgical experience and procedural success.

On the first day, correct identification and looping of both subclavian vessels was achieved in only one third of participants, despite prior surgical experience in many cases. After repetition and supervised reassessment, successful performance increased to more than three quarters of participants, accompanied by a significant reduction in potentially harmful errors. This finding highlights that structured repetition and anatomical familiarity outweigh general surgical experience when performing rare, time-critical procedures.

The absence of correlation between seniority and performance underscores a relevant educational gap. Even experienced trauma surgeons may lack exposure to proximal vascular control of the upper extremity due to the rarity of such injuries. Cadaver-based training therefore represents a safe and effective environment to acquire and rehearse these skills without patient risk.

Recently, Puchwein et al. demonstrated that even unexperienced surgeons and medical students were able to perform an emergency clamshell thoracotomy within 3 min in a cadaver model [14]. Flaris et al. completed a thoracotomy with control of a heart wound within 4–7 min [15]. In Hohenberger et al., Monaldi’s approach for decompression of the tension pneumothorax was performed bilaterally in 82 torsos by six participants with varying training levels (including one student) following instruction by an experienced thoracic surgeon [16]. 83% of all punctures were placed in the correct intercostal space and the post-puncture evaluation did not reveal any iatrogenic injuries to thoracic organs or major vessels. Up until now, the emergency approach to the subclavian vessels represents a novel contribution to the literature.

In the Advanced Trauma Life Support (ATLS^®^) program, there is a clear demand for mastery in definitive hemorrhage control for patients in physiological extremis [9]. However, increasing sub-specialization means that the specific skills required for proximal extremity and trunk bleeding are often infrequently available, as traditional surgical training has become more organ specific. To address this, trauma network strategies in regions like Germany and Austria have focused on implementing specific knowledge to prevent treatment errors [10, 11]. Organizations like the European Society for Trauma and Emergency Surgery therefore promote cadaveric workshops to bridge this gap; these sessions are highly valued for improving technical proficiency and self-confidence where “real practice” is otherwise difficult to obtain [17, 18, 19].

Ultimately, the trauma team leader must be prepared to initiate life-saving damage control vascular surgery when a full multidisciplinary team is not immediately available. The main problem in these unusual situations is that decision-making is performed under acute clinical urgency.

As a limitation, this study was conducted using a cadaveric model, which inherently involves a lack of dynamic physiological factors like scenarios with active hemorrhage and physiological stress. This also includes the changes of cadaveric tissue post-mortem such the absence of tissue perfusion and collapsed vessels. Another factor is the preservation, which can alter the tissue texture, color and elasticity. The controlled educational setting may have influenced participant performance. The absence of clinical pressure, potential complications, unexpected events as well as distractions in a controlled educational setting also represents a limitation. The performance might be different, because the participants knew, that they were observed and evaluated during the procedure. On the second day, reassessment of a previously performed approach likely contributed to improved accuracy. Furthermore, the study focused on anatomical access and vessel identification rather than clinical outcomes, which limits direct conclusions regarding patient benefit. However, cadaveric models are anatomically one of the best techniques to simulate a real-life situation.

## Conclusion

Emergency exposure of the subclavian vessels is challenging for non-thoracic surgeons. No correlation was found between overall surgical experience and successful emergency access. Anatomical dissection training significantly improved both procedural success and confidence among participants who successfully achieved correct vessel clamping, increasing from 27% (9/33) on day one to 71% (55/77) on day two. These findings suggest that accurate self-assessment can be developed through structured anatomical training. Future studies should evaluate how such training models translate into clinical performance and patient outcomes.

## Data Availability

The datasets used and/or analyzed during the current study are available from the corresponding author on reasonable request.

## Abbreviations

e.g.: Exempli gratia
fig.: Figure
etc.: Et cetera
et al.: Et alia
AO: Arbeitsgemeinschaft für Osteosynthesenfragen
SD: Standard deviation
cm: Centimeter
ATLS: Advanced Trauma Life Support
